# Supplementary material for: Direct observation of organic molecules in asteroid ryugu revealed by high-resolution atomic force microscope
Source: Nat Commun. 2026 Apr 14;17:3416. doi: 10.1038/s41467-026-71484-y (PMC13079723; doi:10.1038/s41467-026-71484-y)
Supplement: Supplementary file 1 — Supplementary Information [file 41467_2026_71484_MOESM1_ESM.pdf]

Supplementary Information for "Direct observation of Molecules in Asteroid Ryugu Reveled by High-Resolution Atomic Force Microscope"

Kota Iwata[1*], Yasuhiro Oba[2], Hiroshi Naraoka[3], Hikaru Yabuta[4, 5], Shogo Tachibana[6, 7], Yoshiaki Sugimoto[1, 5*]

[1] *Department of Advanced Materials Science, University of Tokyo, Kashiwa, 277-8561, Japan.*

[2] *Institute of Low Temperature Science, Hokkaido University, Sapporo, 060-0189, Japan.*

[3] *Department of Earth and Planetary Sciences, Kyushu University, Fukuoka, 819-0395, Japan.*

[4] *Department of Earth and Planetary System Science, Hiroshima University, Higashi-Hiroshima, 739-8526, Japan.*

[5] *International Institute for Sustainability with Knotted Chiral Meta Matter (WPI-SKCM²), Hiroshima University, Higashi-Hiroshima, 739-8526, Japan.*

[6] *Department of Earth and Planetary Science, University of Tokyo, Tokyo, 113-0033, Japan.*

[7] *Institute of Space and Astronautical Science, Japan Aerospace Exploration Agency (JAXA), Sagamihara, 252-5210, Japan.*

*Correspondence to: kiwata@g.ecc.u-tokyo.ac.jp; ysugimoto@k.u-tokyo.ac.jp

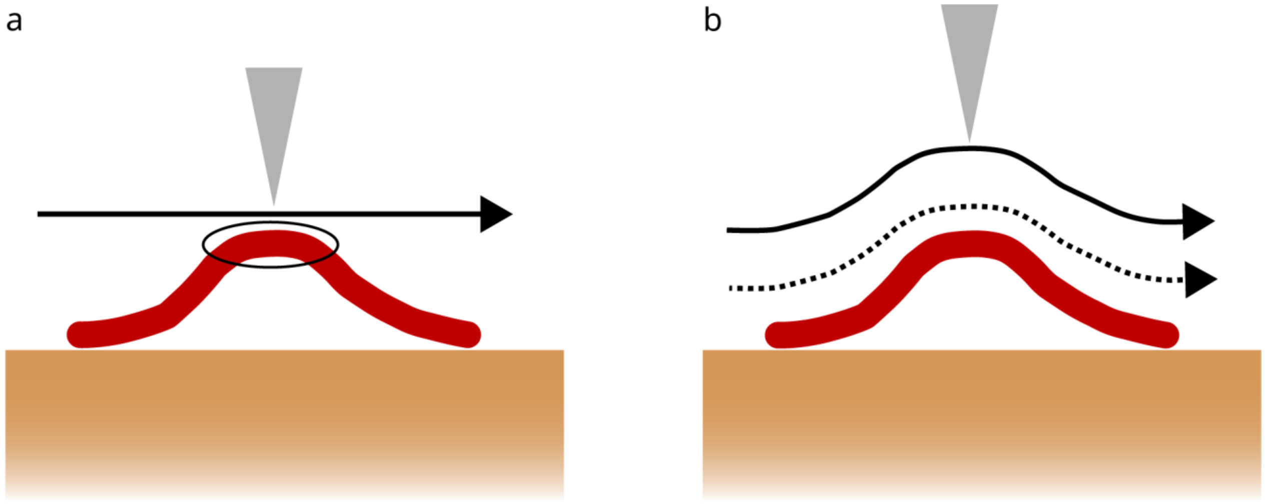

Supplementary Fig. 1 | Schematic illustrations of high-resolution AFM imaging. a. Constant-height mode and **b.** multi-pass mode is schematically illustrated. The thick red lines, grey triangles, and solid and dotted black arrows represent a three-dimensional molecule adsorbed on the substrate, the AFM tips, and their scan trajectories, respectively. In the constant-height mode, the tip scans parallel to the substrate at the fixed tip height, as shown by the arrow. Only the protruding part of the molecule (indicated by a circle) is imaged, because the tip can approach close enough to detect the repulsive force responsible for the high-resolution imaging. In contrast, the tip is difficult to reach the repulsive region over the lower part of the molecule without crashing higher part. In the multi-pass mode, the first scan (solid arrow) is performed with STM feedback control active. Then the second scan (dotted arrow) follows the trajectory of the first scan but with an offset tip height. This mode allows the tip to get close enough to probe the repulsive force even over the lower part of the corrugated molecules.
